# Everybody's Page

**Published:** 1891-07-25

**Authors:** 


### EVERYBODY'S PAGE.

IT is stated that a process has now been discovered for the liquefaction and utilisation for numerous purposes of the carbonic acid gas which is given off largely during the process of fermentation in brewing. The result of this utilisation of what has hitherto been a waste product will help to swell the coffers of the brewers; it should also make aerated waters cheaper.

SOME remarkable reports come from Chili, which mark that country out as anything but a desirable place at the present time in which to be domiciled. We have to go back to the worst days of the Inquisition for a parallel to the iniquities which are said to be perpetrated. Women are reported to be imprisoned daily, and to have nothing to wear, and little to eat, and prisoners to be made to walk on red-hot irons, while pins and needles are driven into their flesh.

THERE is a prospect that in Canada what has been a great injustice to women throughout the British Empire, will shortly be rectified. A Bill has been read a first time in the Senate, proposing to grant a divorce to either party for infidelity, and to the wife for her husband's desertion without sufficient cause for five years. Everything has latterly been tending to the equality of the two sexes, but much still remains to be done in this country, where precedent still holds such sway.

AN unpleasant, if not alarming, prospect is held out to the inhabitants of the West-end of London. The Woolwich Board of Health are reported to have sent an urgent protest to the London County Council upon the foul state of the river Thames, notwithstanding all that has been done during recent years in the way of deodorisation. The water is getting polluted over such a large area, that it is expected to become as badly contaminated at Westminster within a few months as it was before the main drainage works were carried out.

MUSICAL instrument makers have long been in search of a varnish which will produce a mellow sound. The British Consul at Hankow very thoughtfully suggests to them a trial of Chinese varnish. This is the product of a gum which is collected from a tree. Its manufacture is surrounded with charms. It only takes a month to dry, during which time it is poisonous to the eyes. The collection of the gum can only take place in the dark, and the gum cannot be strained in the wet, as moisture causes it to solidify, while to simplify matters still further, the varnish must not be used when the atmosphere is dry! Those people who were fortunate enough to be able to spend so much time in elucidating the puzzle "fifteen" might turn their attention to this trade: they would no doubt find it equally engrossing.

A PILGRIMAGE to Mecca is evidently a journey to be avoided. It is alleged that of all the pilgrims leaving Bombay for Mecca and Medina more than a third do not return. Their disappearance does not appear to be caused by the seductive charms of Mecca, but to be due to numerous unpleasant causes. Many of the pilgrims are old, and die from natural causes, others are decimated by starvation and the unsanitary condition of the vessels on which they sail. Many owe their deaths to murder between Jeddah and Mecca. It is said that certain gentlemen called *budmashes*, a playful term we suppose for headsmashers, are regular customers of the pilgrim steamers, on which they find opportunities for choosing as their victims those of their fellow travellers whose demise will prove most productive to them.

STRYCHNINE injected in the usual manner of hypodermic injections has been used lately in Australia with much success in cases of bites from poisonous snakes and spiders.

RUSSIAN firms are said to be manufacturing *brick tea* on a large scale. Brick buns have been far too familiar to us, and if the latest novelty presents no more seductive charm to the palate than the penny delicacy of our railway buffets, may we be saved from its consumption. In the interests of the army, whom destiny points out as the consumers of all compressed products, we trust it will prove a pleasant drink.

A PERSEVERING gentleman, evidently with plenty of time on his hands, and with a taste for experimentalizing, is reported to have kept a daily record of his pulse for a number of years. The reward of his patience has been the discovery that it has two cycles of increase and decrease in rapidity each year. It falls gradually through the spring until midsummer, and rises through the autumn till November or December. A second fall and rise culminating in February then follows.

THE warrior priest of the middle ages is not yet extinct. The Curé of the village of Salces, near Perpignan, objected the other day to the Mayor ordering his church bells to be rung in celebration of the national fête by closing his church doors. The bell-ringer, however, who seems to have been a better citizen than churchman, climbed the tower, and only desisted from ringing his joyful peal when the priest pointed a loaded pistol at his breast and clenched matters in a practical manner by throwing him down the stairs.

HOSPITAL patients in Berlin and Paris are not to be envied, if it is true, as it appears to be, that experiments have been and are being made on patients by inoculating them with cancer lymph. The surgeons who have so operated state in their defence, that in every case the patient so operated upon was past recovery, and that it was necessary for them to select human beings for experiment, inasmuch as none of the lower animals would have been suitable for their purpose. Anti-vivisectionists would very soon and very justly be up in arms if human life were made of such small account in this country.

IT argues distinct weakness of mind to commit suicide because you are unable to do your housework. But this seems to have been the cause of deep melancholy on the part of a woman in Sydney, who had only been married two weeks, which led to her taking immediate steps to withdraw from the turmoils of matrimonial existence. The means she chose were not romantic or such as would probably commend themselves to the youthful but disappointed lover. She swallowed two teaspoonfuls of "Rough on Rats" which, if she was on good terms with her newly wedded husband, proved rough on him.

THE extension of the Parliamentary franchise to women, who already possess local franchises, seems only logical, though the differences of opinion as to its expediency are probably as great as ever. The right of women to vote in the elections of corporations has not been proved by experience to have been disadvantageous to municipal government, and the opinions of women on such subjects as cruelty to children and kindred matters would prove of undoubted value in the House of Commons. Opponents of the Society for Women's Suffrage are unkind enough to hint that the presence of women in the House, which is the ultimate outcome of giving them the franchise, would tend so to lengthen debate as to make business impossible.

